# The effect of combined carprofen and omeprazole administration on gastrointestinal permeability and inflammation in dogs

**DOI:** 10.1111/jvim.15897

**Published:** 2020-09-07

**Authors:** Susan M. Jones, Ann Gaier, Hiroko Enomoto, Patricia Ishii, Rachel Pilla, Josh Price, Jan Suchodolski, Joerg M. Steiner, Mark G. Papich, Kristen Messenger, M. Katherine Tolbert

**Affiliations:** ^1^ College of Veterinary Medicine, Department of Molecular Biomedical Sciences North Carolina State University Raleigh North Carolina USA; ^2^ Department of Small Animal Clinical Sciences, College of Veterinary Medicine and Biomedical Sciences Texas A&M University College Station Texas USA; ^3^ Office of Information and Technology University of Tennessee Knoxville Tennessee USA

**Keywords:** canine, dysbiosis, nonsteroidal anti‐inflammatory drug, proton pump inhibitor

## Abstract

**Background:**

Proton pump inhibitors (eg, omeprazole) commonly are administered concurrently with nonsteroidal anti‐inflammatory drugs (NSAIDs; eg, carprofen) as prophylaxis to decrease the risk of gastrointestinal (GI) injury. However, evidence to support this practice is weak, and it might exacerbate dysbiosis and inflammation.

**Hypothesis/Objectives:**

To evaluate the effect of carprofen alone or combined with omeprazole in dogs. We hypothesized that coadministration of omeprazole and carprofen would significantly increase GI permeability and dysbiosis index (DI) compared to no treatment or carprofen alone.

**Animals:**

Six healthy adult colony beagle dogs.

**Methods:**

Gastrointestinal permeability and inflammation were assessed by serum lipopolysaccharide (LPS) concentration, plasma iohexol concentration, fecal DI, and fecal calprotectin concentration in a prospective, 3‐period design. In the first 7‐day period, dogs received no intervention (baseline). During the 2nd period, dogs received 4 mg/kg of carprofen q24h PO for 7 days. In the 3rd period, dogs received 4 mg/kg of carprofen q24h and 1 mg/kg of omeprazole q12h PO for 7 days. Gastrointestinal permeability testing was performed at the end of each period. Data were analyzed using repeated measures mixed model analysis of variance with Tukey‐Kramer post hoc tests (*P* < .05).

**Results:**

Serum LPS and plasma iohexol concentrations did not differ between treatments. Fecal calprotectin concentrations differed between treatments (*P* = .03). The DI varied over time based on the treatment received (*P* = .03). Coadministration of omeprazole and carprofen significantly increased fecal calprotectin concentration and DI compared to baseline and carprofen alone.

**Conclusions and Clinical Importance:**

Omeprazole prophylaxis induces fecal dysbiosis and increases intestinal inflammatory markers when coadministered with carprofen to otherwise healthy dogs with no other risk factors for GI bleeding.

**Abbreviations**
ANOVA: analysis of variance
DI: dysbiosis index
GI: gastrointestinal
HPLC: high‐performance liquid chromatography
LPS: lipopolysaccharide
NSAID: nonsteroidal anti‐inflammatory drug
PPI: proton pump inhibitor
qPCR: quantitative PCR
SCFA: short chain fatty acid

## 1 INTRODUCTION

Nonsteroidal anti‐inflammatory drugs (NSAIDs) are commonly prescribed to dogs for the treatment of pain and inflammation, often caused by osteoarthritis. The most commonly reported adverse effects of NSAIDs are gastrointestinal (GI) signs such as vomiting and diarrhea.^1^ More severe effects, including GI ulceration and hemorrhage, also can occur. As demonstrated in humans, the pathophysiology of NSAID‐induced small intestinal injury is distinct from that of the stomach. In the stomach, NSAIDs cause mucosal damage primarily by inhibition of cyclooxygenase enzymes, which decreases protective prostaglandins.^2^ However, with NSAID‐induced enteropathy, bacteria are thought to play a major role as evidenced by studies in which gnotobiotic rats only developed NSAID‐induced intestinal ulceration when colonized by commensal bacteria.^3^ Moreover, broad spectrum antibiotics decrease NSAID‐induced intestinal ulceration in rats,^4^ which further emphasizes the important role that bacteria play in the formation of NSAID‐induced intestinal ulcers. Removal of NSAIDs and treatment with proton pump inhibitors (PPIs), such as omeprazole, are recommended for dogs with documented or suspected NSAID‐induced gastric bleeding.^5^ Gastric acid suppressants also commonly are prescribed prophylactically with NSAIDs with the objective of decreasing the risk of GI bleeding associated with NSAID treatment.^6^ However, PPIs can disrupt the intestinal microbiome in dogs,^7^ which may increase susceptibility to NSAID‐induced enteropathy. Indeed, PPIs induce intestinal dysbiosis and exacerbate NSAID‐induced small intestinal ulceration and bleeding in rats.^8^

Despite the common practice of prophylactically administering PPIs to otherwise healthy dogs receiving NSAIDs, no published studies have investigated the effects of this combination on intestinal permeability and inflammation in dogs. Our study objective was to evaluate the effect of the NSAID, carprofen, on the development of increased GI permeability, inflammation, and fecal dysbiosis when administered alone or in combination with the PPI, omeprazole, in dogs. We hypothesized that coadministration of omeprazole and carprofen would significantly increase GI permeability and the dysbiosis index (DI) compared to no treatment or carprofen alone.

## 2 MATERIALS AND METHODS

### 2.1 Study animals

The study was approved by the Institutional Animal Care and Use Committee at North Carolina State University (Protocol # 19‐048‐O). Six healthy adult purpose‐bred beagle dogs from a research colony at North Carolina State University (4 males, 2 females), aged 1.5 to 6 years and weighing 10.0 to 12.0 kg, were used in the study. Dogs were deemed healthy based on lack of a history of GI disease (eg, vomiting, diarrhea, anorexia), normal physical examination, and normal laboratory diagnostic test results (ie, CBC and serum biochemistry profile) obtained within 6 to 15 months of study entry. Dogs were maintained in their original housing throughout the study and their social interaction and feeding schedule were not altered for the study.

### 2.2 Study design

**FIGURE 1:**
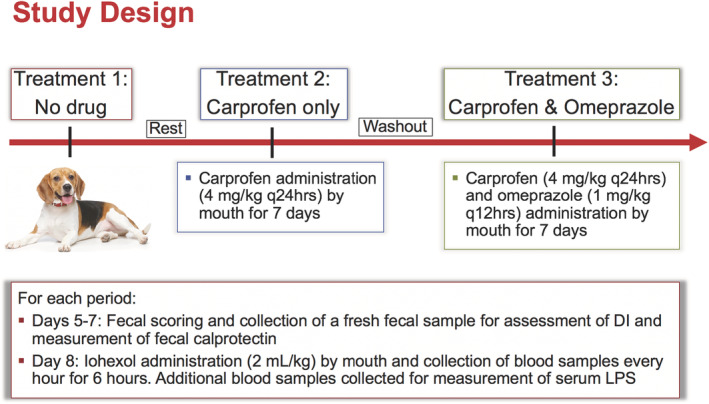
Six beagle dogs were used in a prospective, 3‐period study. “Rest” = 1‐day duration; “Washout” = four‐week duration

A prospective, sequential study was performed (Figure 1) in which dogs received no treatment (baseline), carprofen alone, or carprofen and omeprazole. On the morning of day 0 of each treatment period, dogs were sedated with 13 μg/kg of dexmedetomidine IV (Dexdomitor, 0.5 mg/mL injection, Orion Pharma, Espoo, Finland) to facilitate placement of IV blood sampling catheters into the external jugular vein. The sedation protocol was kept consistent among treatments for each dog. Catheters were sutured in place and covered with a light bandage. During the 1st period (baseline), dogs received no treatment with a similar degree of interaction among dogs and with animal care technicians for 7 days. A 1‐day rest period separated the first and second periods. During the 2nd treatment period, dogs were given 4 mg/kg of carprofen PO q24h for 7 days. After a 4‐week washout period, the same dogs received both carprofen 4 mg/kg q24h PO and omeprazole 1 mg/kg q12h PO for 7 days. All drugs were administered 30 minutes before feeding throughout the study. All dogs were fed their normal diet (Exclusive adult dog food; Land O'Lakes, Inc, Arden Hills, Minnesota) throughout the study. For each period, including baseline, fresh feces were collected in the morning on days 5 to 7 and immediately stored at −80°C. On day 8, food was withheld from the dogs and 2.0 mL/kg of iohexol (Omnipaque‐350, GE Healthcare Ireland, Cork, Ireland) was administered PO. Blood samples were collected hourly for 6 hours after iohexol administration and placed into serum separator and lithium heparin anticoagulant tubes on ice. Serum and plasma were separated from blood samples after centrifugation at 3500*g* for 10 minutes and immediately stored at −80°C. Fecal consistency was graded from 1 to 7 on each day of each treatment period using a standardized fecal scoring system (Fecal Scoring System, Nestlé Purina PetCare Company, St. Louis, Missouri). Diarrhea was defined as a fecal score > 4. Clinical signs, including changes in activity, food consumption, vomiting, and number of defecations, were recorded a minimum of q12h. Fecal and serum samples were shipped on dry ice at study completion to the Gastrointestinal Laboratory at Texas A&M University for measurement of serum lipopolysaccharide (LPS) concentration, fecal DI, and fecal calprotectin concentration.

### 2.3 High‐performance liquid chromatography analysis of iohexol

High‐performance liquid chromatography (HPLC) was used to analyze the concentration of iohexol in plasma samples using a method modified from previous studies.^9^ The HPLC system consisted of a quaternary solvent delivery system and an ultraviolet light detector set at a wavelength of 246 nm (Agilent 1200 Series: Agilent Technologies, Wilmington, Delaware). Separation was achieved using a Zorbax Eclipse C18 column (Agilent Technologies). The mobile phase consisted of 5% acetonitrile in water, adjusted to pH of 2.5 using phosphoric acid, at a flow rate of 1 mL/min. A stock solution of iohexol was prepared fresh daily using an analytical reference standard of iohexol diluted in methanol. Iohexol concentrations were determined from calibration curves that were made fresh and run each day that plasma samples were analyzed. The calibration curve was generated from results of 8 canine plasma standards ranging from 0.5 to 100 μg/mL of iohexol and a blank (0 μg/mL) sample. The calibration curves were linear with a coefficient of determination (*r* ^2^) of >0.99, and the back‐calculated concentrations were within 15% of the true concentration of the standard. Plasma samples of 400 μL were loaded onto washed solid‐phase extraction cartridges (Oasis HLB 1 mL; Waters Corporation, Milford, Massachusetts) and eluted with 1 mL of methanol with 0.25% phosphoric acid. The eluent was evaporated in a water bath at 55°C. The samples were reconstituted with 200 μL of the mobile phase, vortexed, and transferred into the HPLC injection vial. The injection volume was 40 μL.

The assay met system suitability requirements. Iohexol undergoes endo‐exo isomerism and each peak was resolved as the endo‐ (minor) and exo‐ (major) isomer with the endo‐isomer eluting at approximately 4 minutes and the exo‐isomer eluting at approximately 4.8 minutes. For our results, we added the concentrations of each isomer and reported the total.

### 2.4 Serum LPS

Serum was collected on day 8 of each treatment period. Endotoxin quantification was performed using Pierce Chromogenic Endotoxin Quant kit (ThermoFisher). Samples were thawed once at room temperature and immediately diluted 1 : 100. Diluted samples were heat‐shocked for 15 minutes at 70°C to inactivate assay inhibitors. The assay was performed according to the manufacturer's instructions with samples, standards, and blanks run in triplicate. The optical density was measured at 405 nm immediately after assay completion, and quantification was calculated using the low standard curve (0.01‐0.1 EU/mL).

### 2.5 Fecal dysbiosis index

Fecal samples were collected on days 5, 6, and 7 of each treatment period for analysis of the fecal DI, which was measured on each sample individually. Sample DNA was extracted using a MoBio PowerSoil DNA isolation kit (MoBio Laboratories), following the manufacturer's instructions. To calculate a PCR‐based DI, quantitative polymerase chain reaction (qPCR) assays were performed for total bacteria, *Faecalibacterium*, *Turicibacter*, *Escherichia coli*, *Streptococcus*, *Blautia*, *Fusobacterium*, and *Clostridium hiranonis* as previously described.^10^ The DI values can range from −10 to 10, with negative values indicating normobiosis and positive values indicating fecal dysbiosis. For the purposes of our study, a value >2 was considered indicative of a dysbiosis, a value between 0 and 2 was considered equivocal, and a value <2 suggested normobiosis.

### 2.6 Fecal calprotectin

Fecal samples were collected on days 5, 6, and 7 of each treatment period for measurement of fecal calprotectin concentrations. Fecal calprotectin concentrations were measured on each sample individually using an analytically validated radioimmunoassay as previously described.^11^ Data were reported as untransformed mean ± SD for all dogs and days for each treatment period.

### 2.7 Statistical analysis

A power calculation was not performed before the study. Serum LPS concentration was analyzed for differences between baseline, carprofen alone, and carprofen and omeprazole periods using a single factor repeated measures mixed model analysis of variance (ANOVA). Plasma iohexol concentration, fecal calprotectin concentration, fecal DI, and individual bacterial taxa were analyzed using a 2‐within subject factor repeated measures mixed model ANOVA. Data were tested for effects of treatment, time, and the interaction of treatment and time. Dog, dog*treatment, and dog*time were evaluated as random effects and considered in each model. An unstructured Kronecker product covariance structure was applied to each 2‐within subject factor mixed model ANOVA. A Shapiro‐Wilk test for normality and QQ plots were used to evaluate normality of ANOVA residuals. Levene's equality of variances test was used to evaluate equality of treatment variances. Box plots and Studentized residual diagnostics were performed to evaluate each model for the presence of outliers. Log transformations were required for plasma iohexol and fecal calprotectin concentrations in order to meet underlying statistical assumptions. To control for type I error, Tukey's *P* value adjustment was applied to post hoc tests for all analyses. Statistical significance was defined as *P* < .05. All statistical analyses were performed using commercial software (SAS software, version 9.4, Cary, North Carolina, Release TS1M6).

## 3 RESULTS

### 3.1 Serum LPS

**FIGURE 2:**
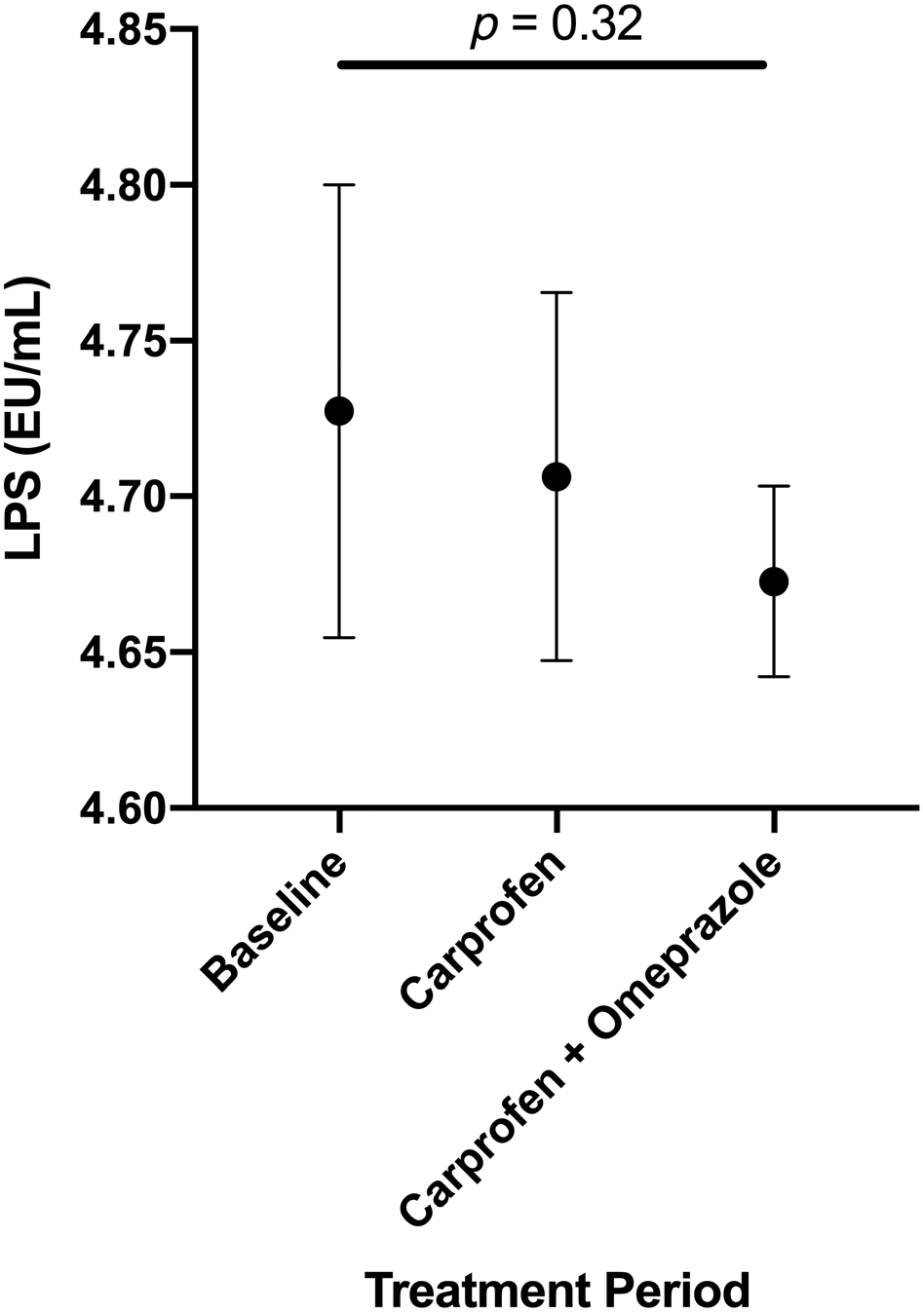
Mean ± SD serum LPS levels (EU/mL) for all dogs receiving no treatment (baseline), carprofen, or carprofen and omeprazole 1 day following each treatment period (day 8). Serum LPS did not differ significantly among treatment periods (*P* = .32). LPS, lipopolysaccharide

Serum LPS concentrations for each treatment are graphically depicted in Figure 2. No significant differences in serum LPS were found among treatments (*P* = .32).

### 3.2 Plasma iohexol

**FIGURE 3:**
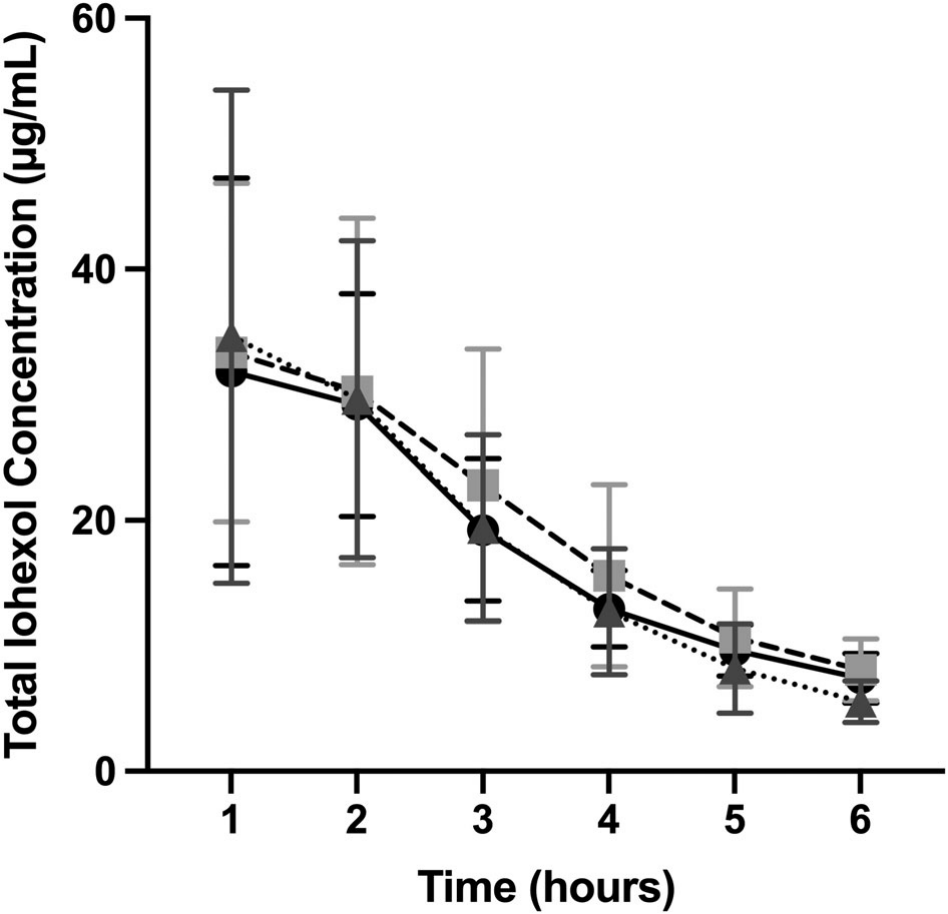
The untransformed mean ± SD total serum iohexol concentration (μg/mL) for all dogs receiving no treatment (baseline, black circles, solid line), carprofen (light gray squares, dashed line), or carprofen and omeprazole (dark gray triangles, dotted line) at hours 1 to 6 one day following each treatment period (day 8). Statistically significant differences were observed over time regardless of treatment received (*P* < .001). Post hoc tests revealed a significant decrease at hours 2 through 6 (*P* < .001, for each). There were no statistically significant differences among treatment periods or treatments over time (*P* = .72 and *P* = .45, respectively)

Total plasma iohexol concentrations (combined endo‐ and exo‐isomer) over time for each treatment are graphically depicted in Figure 3. Significant differences in total plasma iohexol concentrations were observed over hours 1 to 6 regardless of treatment received (*P* < .001). Post hoc tests identified no differences between hours 1 and 2 (*P* = .72). However, for hours 2 through 6, each subsequent time measurement demonstrated a significant decrease in total plasma iohexol concentration (*P* < .001, for each). No significant differences in total plasma iohexol concentration were found between treatments or treatment‐by‐time (*P* = .72 and *P* = .45, respectively).

### 3.3 Fecal dysbiosis index

**FIGURE 4:**
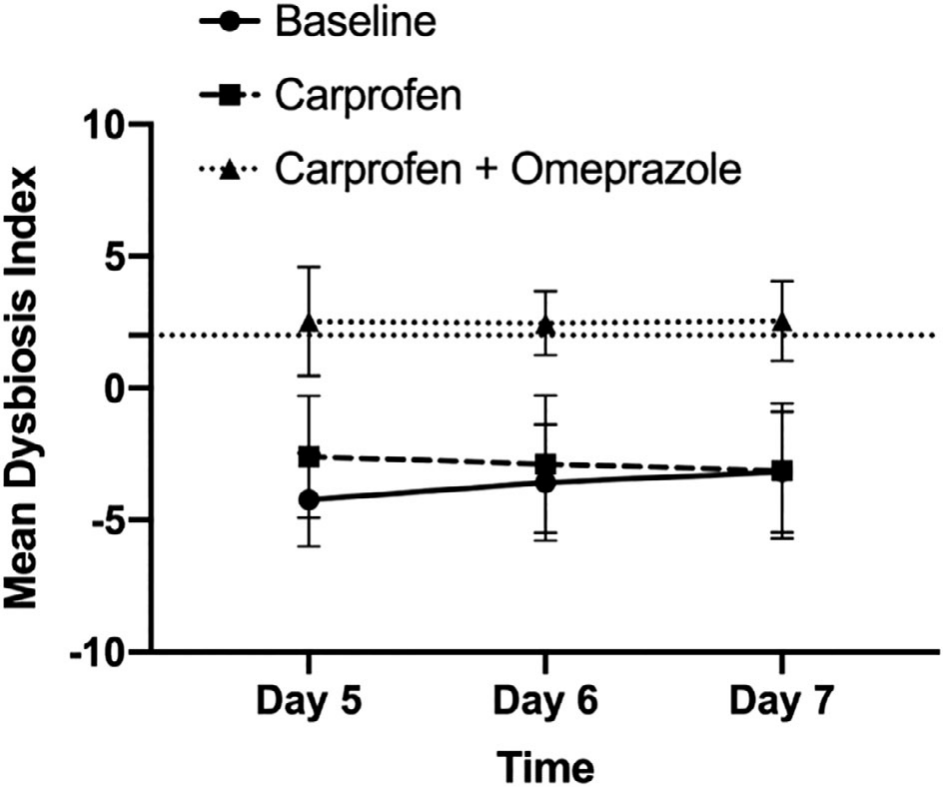
Mean DI ± SD for all dogs receiving no treatment (baseline, circles), carprofen (squares), or carprofen and omeprazole (triangles) over treatment days 5 to 7. Values below zero indicate normobiosis and values above 2 (dotted horizontal line) indicate fecal dysbiosis. A value of 0 to 2 represents a gray zone. The DI significantly varied over time based on the treatment received (*P* = .03). However, after adjusting post hoc tests for multiple comparisons, only differences between treatments were significant. On days 5, 6, and 7, the DI during the coadministration of carprofen and omeprazole differed significantly from baseline and from the period of carprofen administration alone (*P* < .002, for each). No differences were observed when the DI for the carprofen only period was compared to baseline on any day (*P* > .2, for each). DI, dysbiosis index

The fecal DI (Figure 4) varied significantly over time based on the treatment received (*P* = .03). However, after adjusting post hoc tests for multiple comparisons, only differences between treatments at each time point were found to be significant. On days 5, 6, and 7, coadministration of carprofen and omeprazole differed from both carprofen and baseline (*P* < .002, for each). All dogs had increased DI when receiving both omeprazole and carprofen. No significant differences were observed when carprofen alone was compared to baseline on any day (*P* > .24). Fecal DI was increased >2 in 5 of 6 dogs on at least 1 treatment day when receiving coadministered omeprazole and carprofen. In the remaining dog, fecal DI was in the equivocal zone of 0 to 2, with a median value of 1.4. In contrast, fecal DI did not increase >0 on any treatment day when dogs received carprofen alone or no treatment. When individual bacterial taxa were considered (Figure S1 **)**, *Turicibacter*, *Fusobacterium*, and *C hiranonis* abundances differed between treatments (*P* < .001, *P* = .02, and *P* < .001, respectively). Post hoc tests determined that coadministration of carprofen and omeprazole resulted in significantly lower average abundances when compared to both carprofen alone and baseline (*P* < .04, for each). No differences were observed between carprofen alone and baseline on any day (*P* ≥ .11, for each). *Faecalibacterium* abundances varied over time based on the treatment received (*P* = .04), but after adjusting post hoc tests for multiple comparisons, only differences between treatments at each time point were found to be significant. On days 5, 6, and 7, coadministration of carprofen and omeprazole resulted in lower average values compared to carprofen alone or baseline (*P* < .01, for each). No difference was found between carprofen and baseline on any day (*P* > .17, for each). *Blautia* results varied over time based on the treatment received (*P* = .002). On day 5, coadministration of carprofen and omeprazole differed from both carprofen alone and baseline (*P* < .001, for each). On day 6, after adjusting post hoc tests, no differences were observed between coadministration of carprofen and omeprazole and baseline (*P* = .05). However, carprofen alone differed between both baseline and coadministration of carprofen and omeprazole (*P* = .01 and *P* = .001, respectively). On day 7, all treatments differed (*P* ≤ .01, for each) and, as with day 5, coadministration of carprofen and omeprazole resulted in lower average *Blautia* abundances compared to that for carprofen alone or baseline. No differences were observed over time or between treatments for *Streptococcus* or *E coli* (*P* ≥ .16 and *P* ≥ .11, respectively).

### 3.4 Fecal calprotectin

**FIGURE 5:**
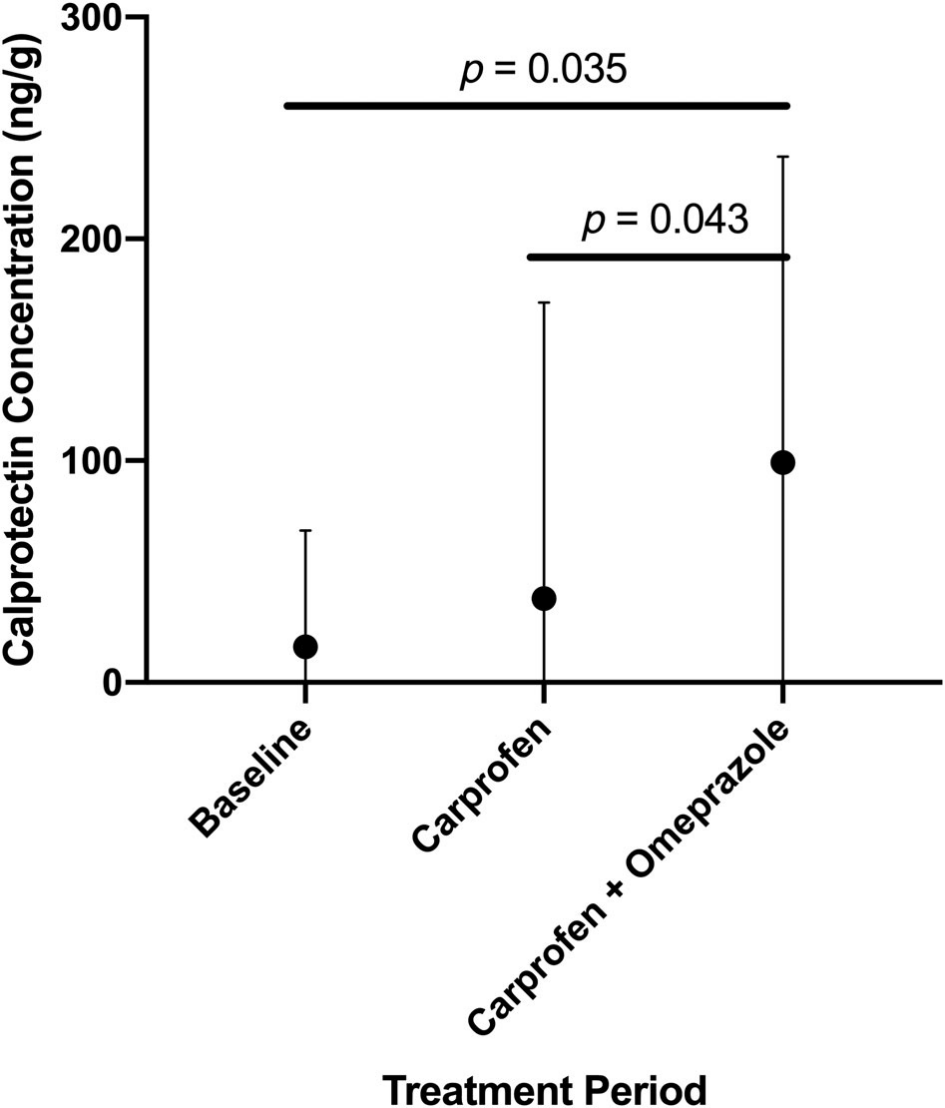
The untransformed mean ± SD fecal calprotectin concentrations (ng/g) for all dogs receiving no treatment (baseline), carprofen, or carprofen and omeprazole averaged over 3 treatment days (days 5‐7). Fecal calprotectin varied by treatment (*P* = .03). The administration of carprofen and omeprazole resulted in significantly increased fecal calprotectin concentrations compared to baseline or carprofen alone (*P* = .04 and *P* = .04, respectively)

Fecal calprotectin concentrations (Figure 5) significantly differed between treatments (*P* = .03) but did not vary over time (*P* = .4), or treatment‐by‐time (*P* = .35). Post hoc tests determined that coadministration of omeprazole and carprofen resulted in increased fecal calprotectin concentrations when compared to baseline (*P* = .03) or carprofen alone (*P* = .04). No differences were observed when carprofen was compared to baseline (*P* = .46).

### 3.5 Adverse effects of treatment

All dogs remained active and alert throughout the study. They all maintained strong appetites and consumed 100% of the food offered during each treatment period. One episode of vomiting during baseline treatment, none during the carprofen treatment, and 3 episodes involving 2 dogs occurred during the carprofen and omeprazole treatment period. Fecal scores remained between 2 and 4 during the baseline and carprofen treatment periods, with averages of 2.8 and 2.5, respectively, for days 5 to 7. During the carprofen and omeprazole treatment period, 3 episodes of diarrhea occurred involving 1 dog, and the average fecal score between days 5 and 7 increased to 3.3. This increase was not statistically significant. No blood was observed in the feces at any time during the study based on macroscopic examination.

## 4 DISCUSSION

We evaluated the effect of carprofen on GI permeability, inflammation, and fecal dysbiosis when administered alone or in combination with omeprazole in healthy dogs. Plasma iohexol and serum LPS concentrations were utilized as markers of intestinal permeability. Iohexol is a large osmotic agent that passively diffuses between epithelial cells throughout the entire GI tract and has been used previously for evaluation of intestinal permeability in healthy dogs.^12^, ^13^ Increased intestinal permeability allows more diffusion and increased appearance of iohexol in the blood. In contrast to other intestinal permeability probes, iohexol is nonradioactive, widely available, and is not degraded by intestinal bacteria. Furthermore, it has been shown to be a reliable substitute for the gold standard of intestinal permeability, Cr‐EDTA.^13^ No significant differences were noted in plasma iohexol concentrations among the treatment periods in our study. Lipopolysaccharide, or endotoxin, is a component of the cell wall of Gram‐negative bacteria in the intestinal tract. Severe loss of intestinal mucosal integrity can increase translocation of LPS and its appearance in the bloodstream as found after NSAID treatment in rats.^14^ However, in agreement with the iohexol data, no significant differences were found in serum LPS concentrations among treatments, suggesting that the short‐term coadministration of omeprazole and carprofen did not significantly increase intestinal permeability in these healthy dogs.

The fecal DI and calprotectin concentrations significantly increased during coadministration of carprofen and omeprazole compared to both the baseline and carprofen alone periods. The fecal DI uses a panel of qPCR assays that evaluate the abundance of 7 key bacterial taxa for intestinal health and enumerates them as a single number.^10^ Fecal DI is often increased in dogs with chronic enteropathies,^10^, ^15^, ^16^ exocrine pancreatic insufficiency,^17^ and during antibiotic administration.^18^ A value >2 indicates the presence of a dysbiosis, a value between 0 and 2 is considered equivocal, and a value <2 suggests normobiosis. The fecal DI was increased >2 in 5 of 6 dogs and >0 in the remaining dog when carprofen and omeprazole were coadministered. In contrast, all dogs receiving no treatment or carprofen alone had a fecal DI < 0. The increase in fecal DI when dogs were given carprofen and omeprazole PO suggests that addition of omeprazole had a negative impact on the fecal microbiome, and in turn may negatively impact intestinal health.

When individual bacterial taxa of the DI were analyzed, the combination of carprofen and omeprazole affected 5 of the 7 key taxa evaluated. Short chain fatty acid (SCFA)‐producing bacteria were found to be decreased after coadministration of omeprazole and carprofen. The SCFA, especially butyrate, are essential for intestinal health,^19^, ^20^ and the decrease in abundance of SCFA‐producing bacteria in the feces of dogs is associated with chronic enteropathy^16^ and acute diarrhea.^21^ The abundance of *C hiranonis*, a bacterium with bile acid 7‐alpha‐dehydroxylation ability,^22^ is quantified in the fecal DI for dogs.^10^ We observed that the addition of omeprazole treatment significantly decreased the abundance of *C hiranonis*. Secondary bile acid production is a key microbiome function known to be impaired after antibiotic administration^18^ and with chronic enteropathies^16^, ^23^ in dogs. It also is believed that lower secondary bile acid concentrations are a predisposing factor for *Clostridioides difficile* infections in humans.^24^, ^25^ In dogs, the pathogenicity of *C difficile* is less clear,^26^, ^27^ but *C hiranonis* may confer resistance to infection.^28^

Calprotectin is a protein complex found primarily in neutrophils and is used as a noninvasive marker of intestinal inflammation in humans and dogs.^29^, ^30^ Fecal calprotectin concentrations can be significantly increased in dogs with chronic enteropathies.^31^, ^32^ In our study, the combination of carprofen and omeprazole significantly increased fecal calprotectin concentrations compared to baseline or carprofen alone, suggesting that addition of omeprazole plays a major role in the development of intestinal inflammation in dogs receiving carprofen.

Whether carprofen plays a substantial role in the induction of dysbiosis or inflammation when coadministered with omeprazole cannot be determined from our study because we did not include a treatment group in which dogs received omeprazole alone. Additionally, our study had a sequential period rather than a randomized design, in which carprofen was administered first to all dogs separated by a 4‐week washout period and followed by coadministration of omeprazole and carprofen. The half‐life of carprofen is approximately 8 hours and 99.9% of the drug is eliminated within 80 hours after administration.^33^ Carprofen‐induced injury to the gastroduodenal mucosa is generally mild and resolves quickly after drug discontinuation.^34^, ^35^ However, we cannot discount the possibility that the order in which the dogs received treatment impacted our results. Based on previous studies in which omeprazole caused intestinal dysbiosis in dogs^7^ and the lack of dysbiosis or inflammation with carprofen alone in our study, we believe that omeprazole is the major driver of fecal dysbiosis and intestinal inflammation and, although further study is needed, may result in development of NSAID‐induced enteropathy, as previously determined in other species.^8^

Our study was performed using 6 healthy beagle dogs with a 7‐day treatment period. Despite the relatively short treatment period, coadministration of omeprazole and carprofen induced a fecal dysbiosis and increased intestinal inflammation in these dogs. Future studies with a larger sample size, a group receiving only omeprazole, and a longer treatment period in a population of dogs with additional risk factors for GI injury and bleeding, such as older age and presence of comorbidities, would better represent the clinical population of dogs receiving carprofen and omeprazole long‐term and may allow for detection of additional changes in GI permeability and determine if any positive benefits from omeprazole administration occur that we were unable to detect. Although such additional studies are warranted, our study results lead us to believe that whereas omeprazole is the standard of care for suspected or documented NSAID‐induced gastric or proximal duodenal bleeding, prophylaxis in healthy dogs receiving carprofen provides no benefit to intestinal health and could cause intestinal inflammation and fecal dysbiosis.

## CONFLICT OF INTEREST DECLARATION

Authors declare no conflict of interest.

## OFF‐LABEL ANTIMICROBIAL DECLARATION

Authors declare no off‐label use of antimicrobials.

## INSTITUTIONAL ANIMAL CARE AND USE COMMITTEE (IACUC) OR OTHER APPROVAL DECLARATION

The IACUC at North Caroline State University approved the protocol for this study.

## HUMAN ETHICS APPROVAL DECLARATION

Authors declare human ethics approval was not needed for this study.

## Supporting information


**Supplementary Figure 1** Bacterial taxa analyzed by qPCR. Turicibacter, Fusobacterium, and C. hiranonis abundances differed between treatments (*P* < .001, *P* = .02, and *P* < .001, respectively). The co‐administration of carprofen and omeprazole resulted in significantly lower average abundances of these bacterial taxa when compared to both carprofen alone and baseline (*P* < .04, for each). Faecalibacterium abundances varied over time based on the treatment received (*P* = .04), but after adjusting post‐hoc tests for multiple comparisons, only differences between treatments at each time point were found to be significant. On days 5, 6, and 7, co‐administration of carprofen and omeprazole resulted in lower average values compared to carprofen alone or baseline (*P* < .01, for each). Blautia values varied over time based on the treatment received (*P* = .002). On days 5 and 7, co‐administration of carprofen and omeprazole resulted in lower average Blautia abundances compared to that for carprofen alone or baseline. No significant differences were observed for Streptococcus or *E. coli* (*P* ≥ .16 and *P* ≥ .11, respectively).
