# Supplementary material for: The effect of combined carprofen and omeprazole administration on gastrointestinal permeability and inflammation in dogs
Source: J Vet Intern Med. 2020 Sep 7;34(5):1886–93. doi: 10.1111/jvim.15897 (PMC7517840; doi:10.1111/jvim.15897)
Supplement: Supplementary file 1 — Supplementary Figure 1 Bacterial taxa analyzed by qPCR. Turicibacter, Fusobacterium, and C. hiranonis abundances differed between treatments (P < .001, P = .02, and P < .001, respectively). The co‐administration of carprofen and omeprazole resulted in significantly lower average abundances of these bacterial taxa when compared to both carprofen alone and baseline (P < .04, for each). Faecalibacterium abundances varied over time based on the treatment received (P = .04), but after adjusting post‐hoc tests for multiple comparisons, only differences between treatments at each time point were found to be significant. On days 5, 6, and 7, co‐administration of carprofen and omeprazole resulted in lower average values compared to carprofen alone or baseline (P < .01, for each). Blautia values varied over time based on the treatment received (P = .002). On days 5 and 7, co‐administration of carprofen and omeprazole resulted in lower average Blautia abundances compared to that for carprofen alone or baseline. No significant differences were observed for Streptococcus or E. coli (P ≥ .16 and P ≥ .11, respectively). [file JVIM-34-1886-s001.pdf]

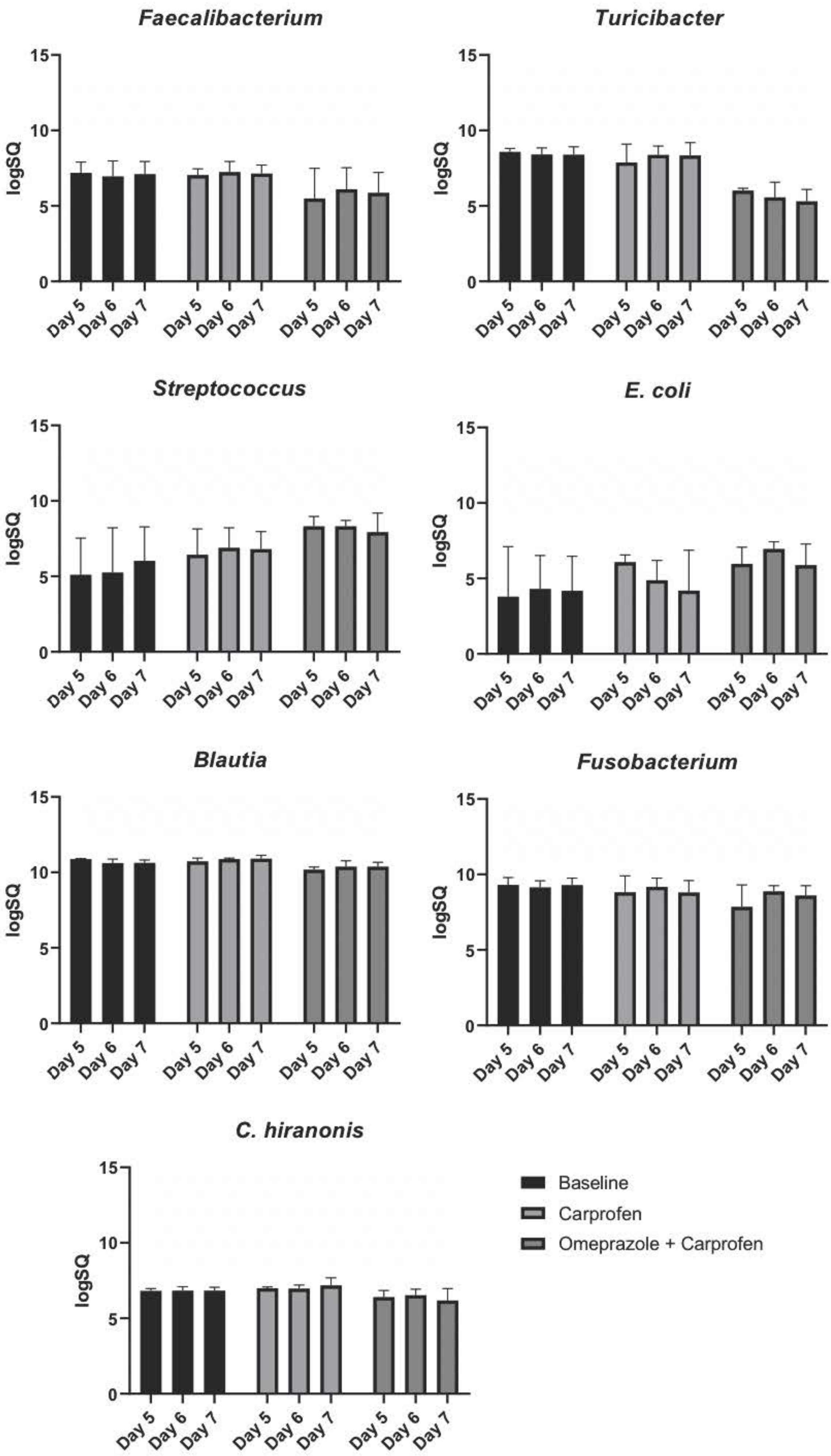

Faecalibacterium
Turicibacter
Streptococcus
E. coli
Blautia
Fusobacterium
C. hiranonis
logSQ
0
5
10
15
Day 5
Day 6
Day 7
Baseline
Carprofen
Omeprazole + Carprofen
